# Mechanisms of tumor necrosis factor-α-induced interleukin-6 synthesis in glioma cells

**DOI:** 10.1186/1742-2094-7-16

**Published:** 2010-03-06

**Authors:** Kumiko Tanabe, Rie Matsushima-Nishiwaki, Shinobu Yamaguchi, Hiroki Iida, Shuji Dohi, Osamu Kozawa

**Affiliations:** 1 Department of Anesthesiology and Pain Medicine, Gifu University Graduate School of Medicine, Gifu 501-1194, Japan; 2 Department of Pharmacology, Gifu University Graduate School of Medicine, Gifu 501-1194, Japan

## Abstract

**Background:**

Interleukin (IL)-6 plays a pivotal role in a variety of CNS functions such as the induction and modulation of reactive astrogliosis, pathological inflammatory responses and neuroprotection. Tumor necrosis factor (TNF)-α induces IL-6 release from rat C6 glioma cells through the inhibitory kappa B (IκB)-nuclear factor kappa B (NFκB) pathway, p38 mitogen-activated protein (MAP) kinase and stress-activated protein kinase (SAPK)/c-*Jun *N-terminal kinase (JNK). The present study investigated the mechanism of TNF-α-induced IL-6 release in more detail than has previously been reported.

**Methods:**

Cultured C6 cells were stimulated by TNF-α. IL-6 release from the cells was measured by an enzyme-linked immunosorbent assay, and the phosphorylation of IκB, NFκB, the MAP kinase superfamily, and signal transducer and activator of transcription (STAT)3 was analyzed by Western blotting. Levels of IL-6 mRNA in cells were evaluated by real-time reverse transcription-polymerase chain reaction.

**Results:**

TNF-α significantly induced phosphorylation of NFκB at Ser 536 and Ser 468, but not at Ser 529 or Ser 276. Wedelolactone, an inhibitor of IκB kinase, suppressed both TNF-α-induced IκB phosphorylation and NFκB phosphorylation at Ser 536 and Ser 468. TNF-α-stimulated increases in IL-6 levels were suppressed by wedelolactone. TNF-α induced phosphorylation of STAT3. The Janus family of tyrosine kinase (JAK) inhibitor I, an inhibitor of JAK 1, 2 and 3, attenuated TNF-α-induced phosphorylation of STAT3 and significantly reduced TNF-α-stimulated IL-6 release. Apocynin, an inhibitor of NADPH oxidase that suppresses intracellular reactive oxygen species, significantly suppressed TNF-α-induced IL-6 release and mRNA expression. However, apocynin failed to affect the phosphorylation of IκB, NFκB, p38 MAP kinase, SAPK/JNK or STAT3.

**Conclusion:**

These results strongly suggest that TNF-α induces IL-6 synthesis through the JAK/STAT3 pathway in addition to p38 MAP kinase and SAPK/JNK in C6 glioma cells, and that phosphorylation of NFκB at Ser 536 and Ser 468, and NADPH oxidase are involved in TNF-α-stimulated IL-6 synthesis.

## Background

Tumor necrosis factor (TNF)-α is a potent pro-inflammatory cytokine with a major role in initiating a cascade of activation of other cytokines and growth factors in inflammatory responses [1]. TNF-α is synthesized by microglia, astrocytes and some populations of neurons and has several important functions in the CNS, including injury-mediated microglial and astrocyte activation, regulation of blood-brain barrier permeability, febrile responses, glutamatergic transmission, and synaptic plasticity [2]. TNF-α leads to activation of inhibitory kappa B (IκB) by the IκB kinase (IKK) complex, which in turn leads to IκB polyubiquitination and subsequent degradation by proteasome [3]. Consequently, nuclear factor kappa B (NFκB) is phosphorylated, liberated from IκB and translocates to the nucleus, where it binds to the promoter regions of NFκB responsive genes and initiates transcription of genes such as those for the proinflammatory cytokines interleukin (IL)-6, IL-1, and TNF-α [1,3]. Each member of NFκB family, such as p65, c-REL, RELB, p105/p50 and p100/p52, can form homodimers, as well as heterodimers with one another. The main activated form of NFκB is a heterodimer of the p65 subunit [1,3]. Different phosphorylation patterns may recruit different transcriptional cofactors to the subunit and induce distinct profiles of gene expression [3]. TNF-α induces IL-6 release through the phosphorylation of NFκB, p38 mitogen-activated protein (MAP) kinase and stress-activated protein kinase (SAPK)/c-*Jun *N-terminal kinase (JNK) in rat C6 glioma cells [4]. TNF-α induces IL-6 expression through the p65 phosphorylation at Ser 276, but not at Ser 529 or Ser 536 in murine fibroblasts [5]. However, the details of NFκB phosphorylation in glial cells have not been clarified.

In addition to the IκB-NFκB pathway, the main intracellular signaling pathway activated by cytokines is the Janus family of tyrosine kinase (JAK)-signal transducer and activator of transcription (STAT) pathway. The activation of the JAK-STAT pathway leads to a rapid signaling from the cell surface to the nucleus [6]. JAK proteins are phosphorylated when cytokines bind to specific receptors, and subsequently activate STATs. The activated STATs translocate to the nucleus and transmit the signals, where they then bind to specific consensus sequences, thereby triggering gene transcription [6]. Seven STAT proteins have been identified in mammalian cells [6]. Among them, STAT1 and STAT3 play important roles in post-ischemic brain damage [7,8]. IL-1β, an important cytokine, phosphorylates STAT3 in C6 cells [9]. However, the precise role of the JAK-STAT pathway in glial cells remains to be elucidated.

Oxidative stress refers to a state with elevated levels of intracellular reactive oxygen species (ROS; such as superoxide radicals and hydrogen peroxide) production and impaired function of antioxidant defense mechanisms. NADPH oxidase is a multi-subunit enzyme that catalyzes the reduction of molecular oxygen and the oxidation of NADPH to generate superoxide radicals [10]. NADPH oxidase is widely distributed and has a variety of functions, such as regulation of immune system, cell growth, cell death and endothelial functions. While NADPH oxidase-derived ROS are necessary for normal cellular functions, excessive oxidative stress can contribute to pathological conditions. ROS play critical roles in TNF-α signaling [11]. NFκB acts as a suppressor of intracellular ROS formation in TNF-α treated cells [11]. Crosstalk occurs between JNK and NFκB, and a role for ROS in TNF-α signaling has emerged. The intermediacy of ROS in the crosstalk between JNK and NFκB is; 1) a TNF-α-induced increase in intracellular ROS is responsible for sustained JNK activation, as well as impaired NFκB activation; 2) NFκB regulates the expression of several key antioxidant enzymes or proteins to eliminate ROS, thus serving as a negative feedback loop; and 3) activated JNK is capable of promoting ROS production, thus forming a positive feedback loop between JNK and ROS [11]. NADPH oxidase in the CNS is associated with memory, neurodegenerative diseases, cerebral ischemic injury and central regulation of the cardiovascular system [10]. NADPH oxidase is found mainly in neurons [12]. Amyloid β induces NADPH oxidase activation and causes oxidative stress in astrocytes [13]. However, the role of NADPH oxidase in astrocytes remains to be fully clarified.

The present study investigated the phosphorylation of specific residues of NFκB is association with TNF-α-stimulated IL-6 synthesis in C6 glioma cells. Furthermore, the involvement of the JAK-STAT pathway and NADPH oxidase in the TNF-α-stimulated IL-6 synthesis was examined.

## Methods

### Materials

TNF-α was obtained from Peprotech (London, UK). IL-6 enzyme-linked immunosolvent assay (ELISA) kit was purchase from R&D System (Minneapolis, MN). Wedelolactone, JAK inhibitor I and apocynin were obtained from Calbiochem-Novabiochem Co. (La Jolla, CA). Phospho-specific IκB, IκB, phospho-specific NFκB (Ser 536, Ser 468 and Ser 276), NFκB, phospho-specific p38 MAP kinase, p38 MAP kinase, phospho-specific SAPK/JNK, SAPK/JNK, phospho-specific STAT3, STAT3 and glyceraldehyde-3-phosphate dehydrogenase (GAPDH) antibodies were purchased from Cell Signaling Technology (Beverly, MA). Phospho-specific NFκB (Ser 529) antibody was purchased from abcam (Cambridge, MA). An enhanced chemiluminescence Western blotting detection system was obtained from GE Healthcare UK. Ltd. (Buckinghamshire, England). Other materials and chemicals were obtained from commercial sources. Wedelolactone and JAK inhibitor I were dissolved in dimethyl sulfoxide. Apocynin was dissolved in ethanol. The maximum concentration of dimethyl sulfoxide or ethanol was 0.1%, which did not affect either the assay for IL-6, the Western blot analysis or the mRNA expression.

### Cell culture

Rat C6 glioma cells, obtained from the American Type Culture Collection (Rockville, MD), were seeded into 35-mm (5 × 10^4 ^cells/dish) or 90-mm (2 × 10^5 ^cells/dish) diameter dishes and maintained in Dulbecco's modified Eagle's medium (DMEM) containing 10% fetal bovine serum at 37°C in a humidified atmosphere of 5% CO_2_/95% air. The medium was exchanged for serum-free DMEM after 6 days. The cells were then used for experiments after 24 h. The cells were pretreated with wedelolactone, JAK inhibitor I or apocynin for 60 min before TNF-α stimulation when indicated.

### Assay for IL-6

Cultured cells (35-mm diameter dishes) were stimulated with 10 ng/ml TNF-α in serum-free DMEM for 36 h. The conditioned medium was collected at the end of the incubation, and IL-6 concentration was measured using an ELISA kit. The absorbance of each sample at 450 nm and 540 nm was measured with a Multiscan JX ELISA reader (Thermo Labsystems, Helsinki, Finland). Absorbance was corrected with reference to a standard curve.

### Western blot analysis

Cultured cells (90-mm diameter dishes) were stimulated with 10 ng/ml TNF-α in serum-free DMEM for the indicated periods. The cells were washed twice with phosphate-buffered saline and then lysed and sonicated in a lysis buffer containing 62.5 mM Tris/HCl (pH 6.8), 2% sodium dodecyl sulfate (SDS), 50 mM dithiothreitol, and 10% glycerol. The sample was used for the Western blotting analysis as described previously [9]. The samples were separated by SDS-polyacrylamide gel electrophoresis by the method of Laemmli [14] in 10% polyacrylamide gels. Western blot analysis was performed using phospho-specific IκB antibodies, IκB antibodies, phospho-specific NFκB antibodies, NFκB antibodies, phospho-specific p38 MAP kinase antibodies, p38 MAP kinase antibodies, phospho-specific SAPK/JNK antibodies, SAPK/JNK antibodies, phospho-specific STAT3 antibodies, STAT3 antibodies or GAPDH antibodies, with peroxidase-labeled antibodies raised in goat against rabbit IgG being used as second antibodies. Peroxidase activity on polyvinylidene difluoride membrane was visualized on X-ray film by utilizing an enhanced chemiluminescence Western blotting detection system.

### Real-time reverse transcription (RT)-polymerase chain reaction (PCR)

Cultured cells (35-mm diameter dishes) were stimulated with 10 ng/ml TNF-α for the indicated periods. Total RNA was isolated and transcribed into complementary DNA (cDNA) using Trizol reagent and Omniscript Reverse Transcriptase Kit. Real-time PCR was performed using a Light Cycler system (Roche Diagnostics, Basel, Switzerland) in the capillaries and Fast-Start DNA Master SYBR Green I provided with the kit. Sense and antisense primers for mouse IL-6 or GAPDH mRNA were purchased from Takara Bio Inc. (Tokyo, Japan) (primer set ID:MA039013). The amplified products were determined by a melting curve analysis and agarose electrophoresis. IL-6 mRNA levels were normalized with those of GAPDH mRNA.

### Statistical analysis

The data were analyzed by ANOVA followed by Bonferroni's method for multiple comparisons between pairs. *P *< 0.05 was considered to be significant. All data are presented as the mean ± SD of triplicate determinations. Each experiment was repeated three times with similar results.

## Results

### Effect of TNF-α on IκB and NFκB phosphorylation in C6 cells

**Figure 1:**
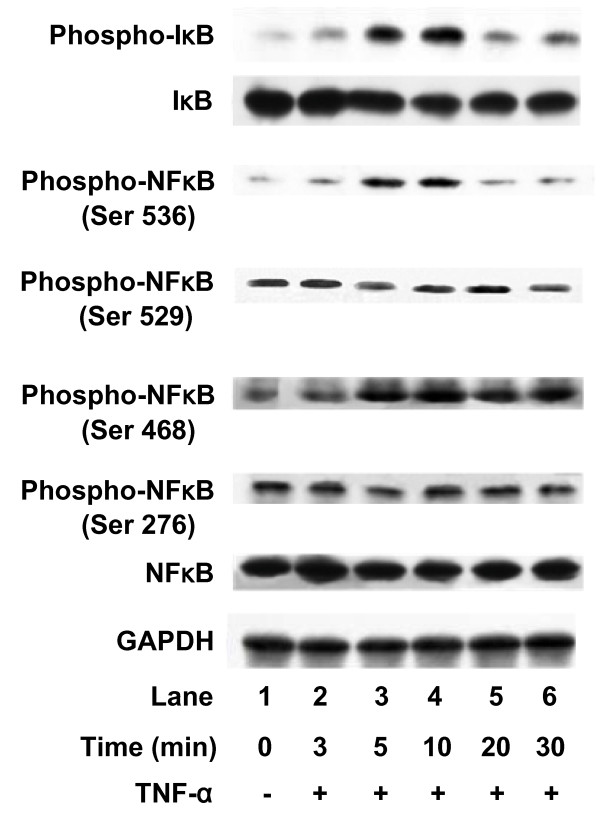
**Effects of TNF-α on IκB phosphorylation and degradation and NFκB phosphorylation**. Cultured cells were stimulated with 10 ng/ml TNF-α for the indicated period. Cell extracts were analyzed by Western blotting using antibodies against phospho-specific IκB, IκB, phospho-specific NFκB, NFκB or GAPDH. Similar results were obtained with two additional and different cell preparations.

The IκB-NFκB pathway is involved in TNF-α-induced IL-6 release from C6 glioma cells [4]. NFκB binds to its consensus sequence on a target gene promoting transcription and upregulation of gene expression [3]. The phosphorylation of NFκB at different sites shows different gene expressions [3]. The effects of TNF-α on the phosphorylation of four serine residues (Ser 536, Ser 529 and Ser 468, Ser 276) were examined. TNF-α significantly induced phosphorylation of NFκB at Ser 536 and Ser 468 in a time-dependent manner. Ser 536 phosphorylation was observed at 5 min and decreased after 20 min, and Ser 468 phosphorylation was enhanced at 5 min and maintained a plateau for up to 30 min (Fig. 1). On the contrary, Ser 529 and Ser 276 were phosphorylated in unstimulated cells, but they were not affected by TNF-α stimulation.

### Effect of wedelolactone on TNF-α-induced NFκB phosphorylation in C6 cells

**Figure 2:**
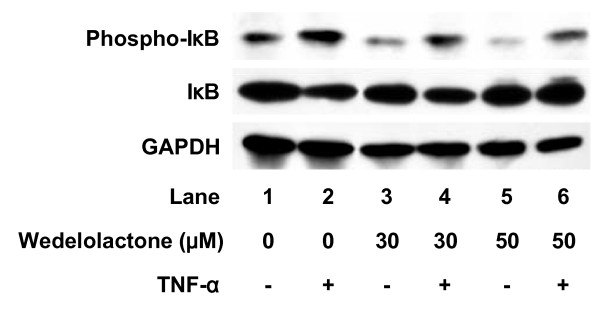
**Effects of wedelolactone on TNF-α-induced phosphorylation and degradation of IκB**. Cultured cells were pretreated with various concentrations of wedelolactone or vehicle for 60 min, and then stimulated with 10 ng/ml TNF-α or vehicle for 10 min. Cell extracts were analyzed by Western blotting using antibodies against phospho-specific IκB, IκB or GAPDH. Similar results were obtained with two additional and different cell preparations.

**Figure 3:**
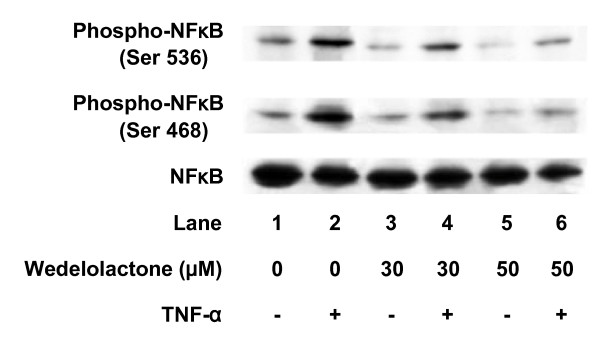
**Effects of wedelolactone on TNF-α-induced phosphorylation of NFκB**. Cultured cells were pretreated with various concentrations of wedelolactone or vehicle for 60 min, and then stimulated with 10 ng/ml TNF-α or vehicle for 10 min. Cell extracts were analyzed by Western blotting using antibodies against phospho-specific NFκB or NFκB. Similar results were obtained with two additional and different cell preparations.

**Figure 4:**
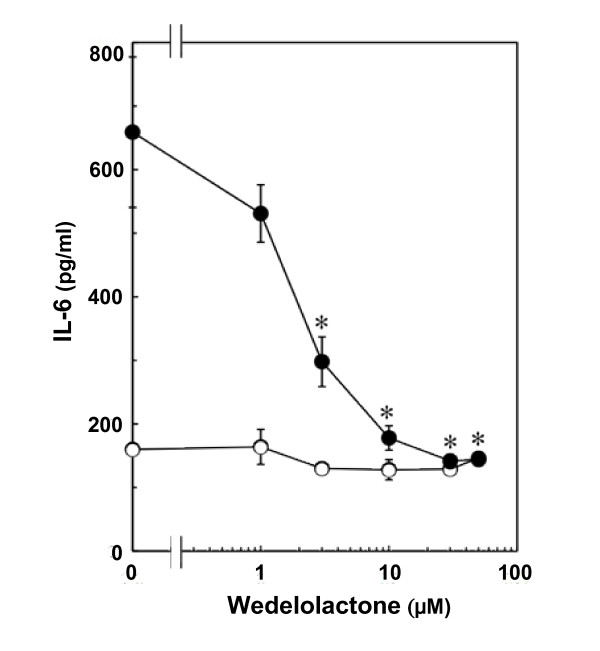
**Effects of wedelolactone on TNF-α-induced IL-6 release**. Cultured cells were pretreated with various concentrations of wedelolactone for 60 min, and then stimulated with 10 ng/ml TNF-α (closed circle) or vehicle (open circle) for 36 h. Each value represents the mean ± SD of triplicate independent determinations of a representative experiment carried out three times. Similar results were obtained with two additional and different cell preparations. *P < 0.05 in comparison to the value of TNF-α alone.

Wedelolactone (30 μM), an inhibitor of IKK [15], suppressed TNF-α-induced IκB phosphorylation (Fig. 2). In addition, phosphorylation of NFκB at both Ser 536 and Ser 468 was inhibited by wedelolactone (Fig. 3). Wedelolactone, which by itself had little effect on the IL-6 levels, significantly suppressed TNF-α-induced IL-6 release. The suppressive effect was concentration-dependent in the range between 1 and 50 μM (Fig. 4).

### Effect of JAK inhibitor I on TNF-α-induced IL-6 release from C6 cells

**Figure 5:**
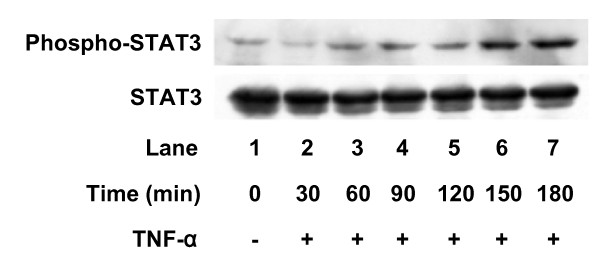
**Effects of TNF-α on STAT3 phosphorylation**. Cultured cells were stimulated with 10 ng/ml TNF-α for the indicated period. Cell extracts were analyzed by Western blotting using antibodies against phospho-specific STAT3 or STAT3. Similar results were obtained with two additional and different cell preparations.

**Figure 6:**
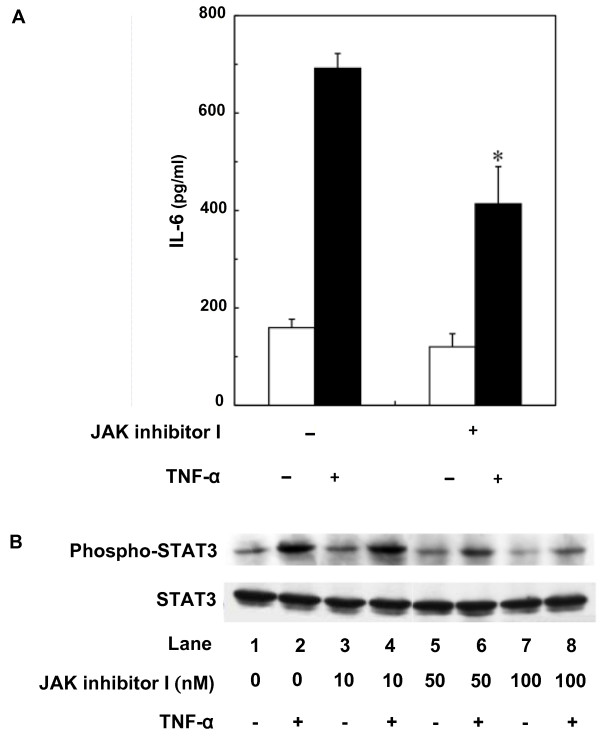
**Effect of JAK inhibitor I on TNF-α-induced IL-6 release**. (A) Cultured cells were pretreated with 10 nM JAK inhibitor I for 60 min, and then stimulated with 10 ng/ml TNF-α or vehicle for 36 h. Each value represents the mean ± SD of triplicate independent determinations of a representative experiment carried out three times. *P < 0.05 in comparison to the value of TNF-α alone. (B) Cultured cells were pretreated with various concentrations JAK inhibitor I or vehicle for 60 min, and then stimulated with 10 ng/ml TNF-α or vehicle for 150 min. Cell extracts were analyzed by Western blotting using antibodies against phospho-specific STAT3 or STAT3. Similar results were obtained with two additional and different cell preparations.

The JAK-STAT pathway has an essential role in driving a variety of biological responses to cytokines [6]. The effect of TNF-α on the phosphorylation of STAT3 was examined. Phosphorylation of STAT3 was marked at 60 min, and reached a peak 150 min after stimulation (Fig. 5). The effect of JAK inhibitor I, an inhibitor of JAK 1, 2 and 3 [16], on TNF-α-induced IL-6 release was examined. JAK inhibitor I (10 nM), which by itself had little effect on the IL-6 levels, significantly suppressed TNF-α-induced IL-6 release (Fig. 6A). In addition, JAK inhibitor I truly suppressed TNF-α-induced phosphorylation of STAT3 in a concentration-dependent manner between 10 and 100 nM (Fig. 6B). The TNF-α-induced phosphorylation of STAT3 was observed later than phosphorylation of IκB, NFκB or MAP kinases. This delayed phosphorylation is consistent with a previous report, showing that IL-1β phosphorylates STAT3 60 min after stimulation in C6 cells [9].

### Effect of apocynin on TNF-α-induced IL-6 release from C6 cells

**Figure 7:**
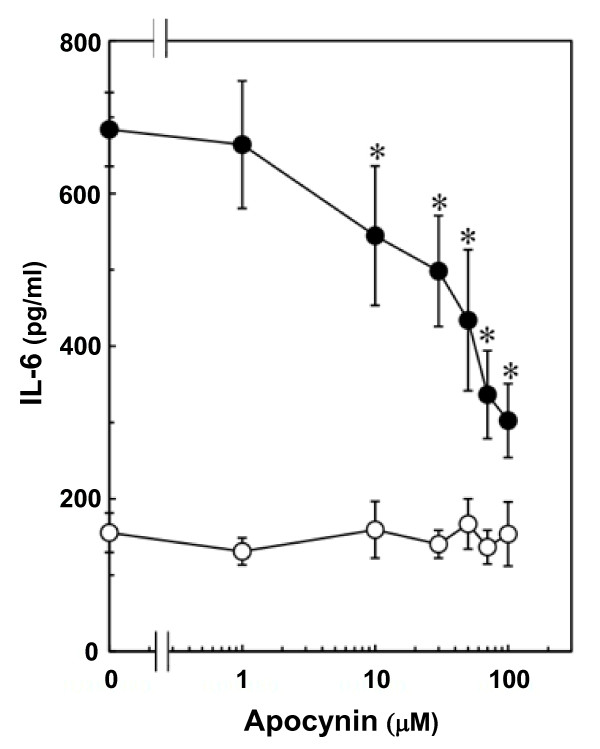
**Effect of apocynin on TNF-α-induced IL-6 release**. Cultured cells were pretreated with various concentrations apocynin for 60 min, and then stimulated with 10 ng/ml TNF-α (closed circle) or vehicle (open circle) for 36 h. Each value represents the mean ± SD of triplicate independent determinations of a representative experiment carried out three times. Similar results were obtained with two additional and different cell preparations. *P < 0.05 in comparison to the value of TNF-α alone.

**Figure 8:**
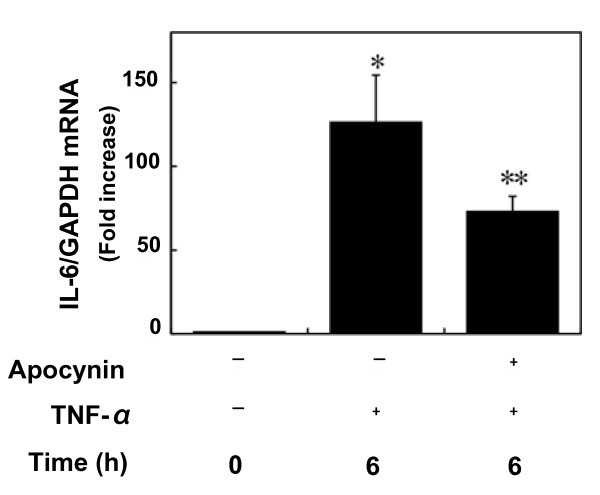
**Effect of apocynin on TNF-α-induced IL-6 mRNA expression**. Cultured cells were pretreated with 0.1 mM apocynin or vehicle for 60 min, and then stimulated with 10 ng/ml TNF-α for 6 h. Total RNA was isolated and transcribed into cDNA. The expressions of IL-6 mRNA and GAPDH mRNA were quantified by real-time RT-PCR. IL-6 mRNA levels were normalized with those of GAPDH mRNA. Each value represents the mean ± SD of triplicate independent determinations of a representative experiment carried out three times. Similar results were obtained with two additional and different cell preparations. *P < 0.05 in comparison to the value of unstimulated cells. **P < 0.05 in comparison to the value of TNF-α alone.

**Figure 9:**
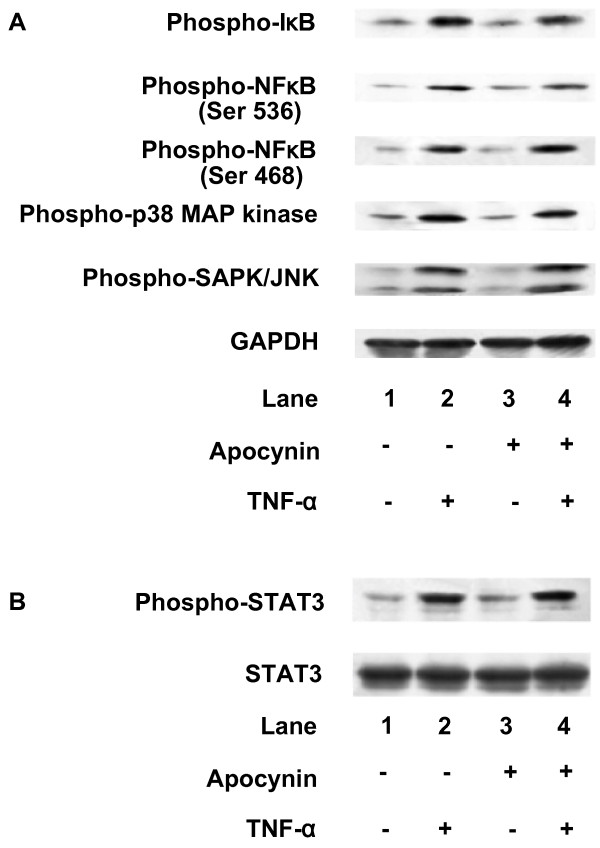
**Effect of apocynin on TNF-α-induced the phosphorylation of IκB, NFκB, MAP kinases and STAT3**. Cultured cells were pretreated with 0.1 mM apocynin for 60 min, and then stimulated with 10 ng/ml TNF-α for 10 min (A) or 150 min (B). Cell extracts were analyzed by Western blotting using antibodies against phospho-specific IκB, phospho-specific NFκB, phospho-specific p38 MAP kinase, phospho-specific SAPK/JNK, GAPDH, phospho-specific STAT3 or STAT3. Similar results were obtained with two additional and different cell preparations.

Apocynin, an inhibitor of NADPH oxidase [17], which by itself had little effect on IL-6 levels, significantly suppressed TNF-α-induced IL-6 release (Fig. 7). This suppressive effect was concentration-dependent in the range between 1 and 100 μM. In addition, TNF-α (10 ng/ml) significantly induced IL-6 mRNA expression at 6 h after stimulation (Fig. 8), thus suggesting that TNF-α stimulates the synthesis of IL-6 in C6 glioma cells. The suppressive effect of apocynin on IL-6 release by TNF-α could be due to protein synthesis suppression. Apocynin (100 μM) suppressed TNF-α-induced IL-6 mRNA expression (Fig. 8). The effects of apocynin on TNF-α-induced phosphorylation of IκB, NFκB, p38 MAP kinase, SAPK/JNK or STAT3 were examined to determine whether the apocynin-effect on TNF-α-induced-IL-6 release is dependent upon activation of the IκB-NFκB pathway, the MAP kinase superfamily, and the JAK-STAT3 pathway in C6 cells. However, apocynin failed to affect the phosphorylation of these kinases (Fig. 9).

## Discussion

TNF-α-induced IL-6 release is dependent on the IκB-NFκB pathway [4]. Several sites in the N-terminal Rel homology domain, including Ser 276, or in the C terminus transactivation domain region play a pivotal role in the finer regulation of NFκB transcriptional activity [3]. Different phosphorylation patterns may induce distinct profiles of gene expression [3]. Therefore, the present study investigated which residue is involved in the TNF-α-stimulated IL-6 synthesis in C6 glioma cells. TNF-α significantly induced the phosphorylation of NFκB at Ser 536 and Ser 468, but not at Ser 529 and Ser 276. Ser 276 phosphorylation by TNF-α is essential for IL-6 production in murine fibroblasts [5]. However, Ser 276 is phosphorylated in unstimulated rat C6 glioma cells and the levels do not change with TNF-α stimulation. Furthermore, wedelolactone, an IKK inhibitor [15], truly reduced the TNF-α-induced IL-6 release and NFκB phosphorylation at Ser 536 and Ser 468. Therefore, these findings suggest that TNF-α induces IL-6 release through phosphorylation of NFκB at Ser 536 and Ser 468 in C6 glioma cells.

JAKs are constitutively associated with many cytokine receptors [6]. The binding of cytokines to a receptor associated with JAKs leads to the tyrosine phosphorylation of the receptor, and generates a docking site for STATs [6]. The STATs are thus phosphorylated and translocate to the nucleus where they may activate transcription of several genes [6]. The present study demonstrated that TNF-α significantly induced phosphorylation of STAT3 in C6 cells, and that JAK inhibitor I, an inhibitor of JAK 1, 2 and 3 [16], suppressed TNF-α-induced IL-6 release. In addition, JAK inhibitor I suppressed TNF-α-induced STAT3 phosphorylation. Therefore, these results suggest that TNF-α induces IL-6 release through the JAK-STAT3 pathway in addition to p38 MAP kinase and SAPK/JNK in C6 glioma cells.

Finally, apocynin, an inhibitor of NADPH oxidase [17], significantly suppressed TNF-α-induced IL-6 release and mRNA expression. The action point of NADPH oxidase in TNF-α-stimulated IL-6 synthesis in C6 glioma cells was investigated. However, apocynin did not affect TNF-α-induced IκB, NFκB, p38 MAP kinase, SAPK/JNK or STAT3 phosphorylation. It therefore seems unlikely that NADPH oxidase functions at a point of these kinases in TNF-α-induced IL-6 synthesis in C6 glioma cells. The current findings are consistent with a previous report, in which ROS was said to be regulated by NFκB and JNK activation [11].

IL-6 plays a key role in B cell maturation and drives acute inflammatory responses [18,19]. IL-6 is produced in neurons, microglia and astrocytes, and it plays a pivotal role in a variety of CNS functions such as induction and modulation of reactive astrogliosis, pathological inflammatory responses and neuroprotection [18,20]. Various stimuli, such as infection, traumatic brain injury, ischemia and CNS diseases produce key inflammatory mediators including IL-6 [18]. TNF-α induces other cytokines and it also plays important roles in ROS production [11]. In addition, NADPH oxidase plays an important role in the immune system in the brain [10]. However, the exact role of NADPH oxidase in astrocytes, not in neurons, remains to be fully clarified. The present study suggests that NADPH oxidase regulates the immune system in the CNS via regulation of IL-6 synthesis in astrocytes.

In conclusion, the current results strongly suggest that TNF-α induces IL-6 synthesis through phosphorylation of NFκB at Ser 536 and Ser 468, and JAK-mediated phosphorylation of STAT3 in addition to p38 MAP kinase and SAPK/JNK in C6 glioma cells, and that oxidative stress is associated with IL-6 synthesis.

## Competing interests

The authors declare that they have no competing interests.

## Authors' contributions

KT and OK conceived the study, participated in its design and coordination, analyzed the data and drafted the manuscript. KT, RM-N and SY performed the experiments. HI and SD provided useful advice. All authors read and approved the final manuscript.
